# The Application of FTIR Spectroscopy and Chemometrics for the Authentication Analysis of Horse Milk

**DOI:** 10.1155/2022/7643959

**Published:** 2022-02-22

**Authors:** Mitsalina Fildzah Arifah, Irnawati, Ruslin, Khoirun Nisa, Anjar Windarsih, Abdul Rohman

**Affiliations:** ^1^Center of Excellence, Institute for Halal Industry and Systems, Universitas Gadjah Mada, Yogyakarta 55281, Indonesia; ^2^Department of Pharmaceutical Chemistry, Faculty of Pharmacy, Universitas Gadjah Mada, Yogyakarta 55281, Indonesia; ^3^Faculty of Pharmacy, Halu Oleo University, Kendari 93232, Indonesia; ^4^Research Division for Natural Product Technology (BPTBA), National Research and Innovation Agency (BRIN), Yogyakarta 55861, Indonesia

## Abstract

Expensive milk such as horse's milk (HM) may be the target of adulteration by other milk such as goat's milk (GM) and cow's milk (CM). FTIR spectroscopy in combination with chemometrics of linear discriminant analysis (LDA) and multivariate calibrations of partial least square regression (PLSR) and principal component regression (PCR) was used for authentication of HM from GM and CM. Milk was directly subjected to attenuated total reflectance (ATR) spectral measurement at midinfrared regions (4000-650 cm^−1^). Results showed that LDA could make clear discrimination between HM and HM adulterated with CM and GM without any misclassification observed. PLSR using 2^nd^ derivative spectra at 3200-2800 and 1300-1000 cm^−1^ provided the best model for the relationship between actual values of GM and FTIR predicted values than PCR. At this condition, *R*^2^ values for calibration and validation models obtained were 0.9995 and 0.9612 with RMSEC and RMSEP values of 0.0093 and 0.0794. PLSR using normal FTIR spectra at 3800-3000 and 1500-1000 cm^−1^ offered *R*^2^ for the relationship between actual values of CM and FTIR predicted values of >0.99 in calibration and validation models with low errors of RMSEC of 0.0164 and RMSEP of 0.0336 during authentication of HM from CM. Therefore, FTIR spectroscopy in combination with LDA and PLSR is an effective method for authentication of HM from GM and CM.

## 1. Introduction

Milk is a good source of protein needed for human development. Milk also contains numerous bioactive molecules, which protect against microbial infection and inflammation and contribute to immune maturation and healthy microbial colonization [1, 2]. Due to price discrepancy, expensive milk was adulterated with cheaper price milk to get economic profits. In the milk industry, horse's milk (HM) is extravagant milk to produce in comparison to cow's milk (GM) and goat's milk (GM). HM is far more nutritious than any other milk, along with CM and GM. HM contains only 44 calories per 100 grams, compared to 64 for cows and 70 for human milk [3]. HM had a similar composition compared to human milk for whey protein and casein, but metabolic profiles examined different HM to human milk [4, 5]. Therefore, HM may be an adulteration target with CM and GM. The adulteration practice of dairy products, involving milk, was typically done by substituting or diluting high price milk with cheaper price milk [6].

Milk authenticity is an important issue nowadays, not only for producers and consumers but also for the regulatory bodies, as consequently, some analytical methods capable of detecting the adulteration practice and quantifying the adulterants are needed [7]. These methods included ultraperformance liquid chromatography-tandem triple quadrupole mass spectrometry (UPLC-TOF MS) by determining the peptide markers [8] and metabolomics approach [9], LC-MS based on ion-trap for proteomics [10] and peptide analyses [11], GC-MS and GC-FID by determining fatty acid composition [12], differential scanning calorimetry (DSC) coupled with machine learning detecting the thermal profile of authentic and adulterated milk [13], and ICP-MS discriminating milk by geographical origin clustering [14]. Chromatographic-based techniques coupled with MS detectors are widely used detection methods, despite these methods being expensive, involving sophisticated instruments, and needing competent analysts. To this difficulty, an easy and reliable technique based on vibrational spectroscopy authenticated milk from adulterated milk.

Vibrational spectroscopy (Raman and infrared spectroscopy), based on the interaction of samples with electromagnetic radiation in the infrared region, is one of the fingerprinting techniques widely reported for the authentication analysis of dairy products, including milk, especially in combination with chemometrics [15]. Chemometrics is the appliance of mathematical and statistical techniques to extract the chemical responses into more understandable information such as pattern recognition patterns and discrimination [16]. Raman spectroscopy and chemometrics of pattern recognition applied for milk authenticity offer a reliable and easy method. Near-infrared was also successful for the authentication analysis of organic milk [17], while raw milk from reconstituted milk was determined using midinfrared [18] and determination of different milk species [19]. Now, reports are available related to the authenticity of horse milk; for this reason, this study is aimed at developing FTIR spectroscopy and chemometrics for authentication analysis of HM from cow milk (CM) and goat milk (GM).

## 2. Materials and Methods

### 2.1. Materials

**Figure 1:**
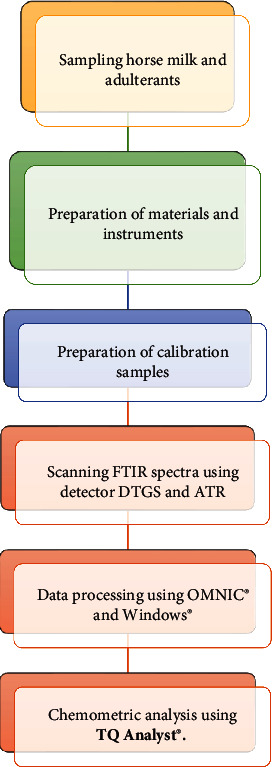
Flow chart for the authentication analysis for horse's milk using FTIR spectroscopy and chemometrics.

The horse's milk samples were collected from a farm in West Nusa Tenggara. Cow milk (CW) and goat milk (GM) were available from farms in Yogyakarta, Indonesia. All samples were stored in a refrigerator at -4°C before being used for analysis. All procedures are shown in Figure 1.

### 2.2. Preparation of Calibration Samples

Calibration samples prepared a set of 75 calibration samples. HM was mixed with CM and GM in the concentration binary mixture range of 0-100%. Validation samples comprising of HM, CM, and GM evaluated the calibration models. FTIR spectral measurement subjected all samples. The composition of HM in a binary mixture with GM as well as HM in a binary mixture with CM is compiled in Table 1.

### 2.3. Linear Discriminant Analysis (LDA)

LDA was used for discrimination between HM and HM adulterated with CM and GM. The samples consisted of pure HM and HM mixed with CM and GM at different concentrations covering 1-100%. Discrimination between authentic and adulterated HM constructed Cooman's plot.

### 2.4. Scanning FTIR Spectra

Spectrophotometer FTIR (FTIR Nicolet iS20) using detector DTGS (deuterated triglycine sulfate) was connected to software OMNIC® and Windows®. The samples were directed placed into multibounce attenuated total reflectance (ATR) crystal, scanned using a resolution of 8 cm^−1^ and number scanning of 64. All spectra were measured at the midinfrared region (4000–650 cm^−1^) using air as background. All spectra were recorded to the absorbance mode to facilitate quantitative analysis according to the Lambert-Beer law. The data obtained was managed using the software of TQ Analyst®.

### 2.5. Chemometrics Analysis

TQ Analyst is used for chemometrics analysis, including LDA and multivariate calibrations (PLSR and PCR). LDA assessed discrimination between authentic and adulterated HM by accuracy levels. In addition, multivariate calibrations were evaluated by the root mean square error of calibration (RMSEC), root mean square error of prediction (RMSEP), and coefficient of determination (*R*^2^).

## 3. Results and Discussion

**Figure 2:**
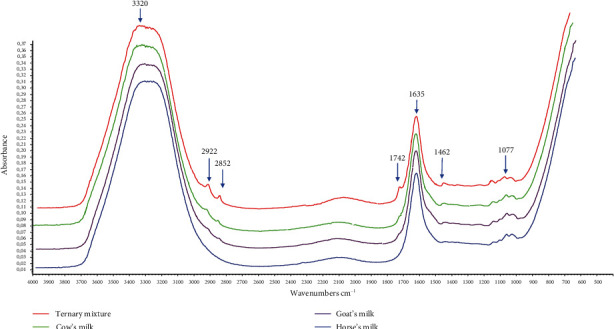
FTIR spectra of pure horse milk (HM), goat milk (GM), cow milk (CM), and ternary mixture (HM, GM, and CM) were scanned using attenuated total reflectance (ATR) mode in the infrared region (4000-650 cm^−1^).

**Figure 3:**
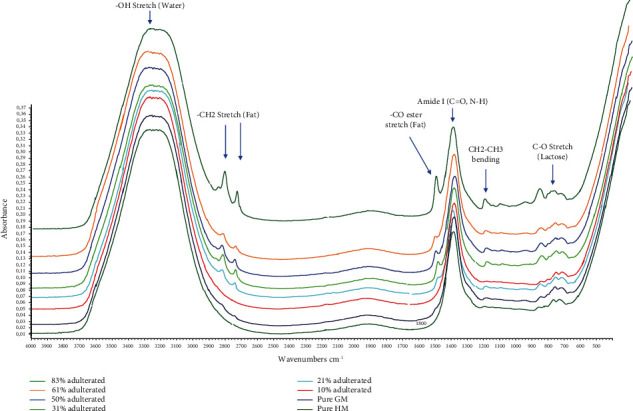
FTIR spectra of the binary mixture were selected from pure horse's milk (HM), goat's milk (GM), and adulterated milk (HM-GM) in the infrared region (4000-650 cm^−1^).

**Figure 4:**
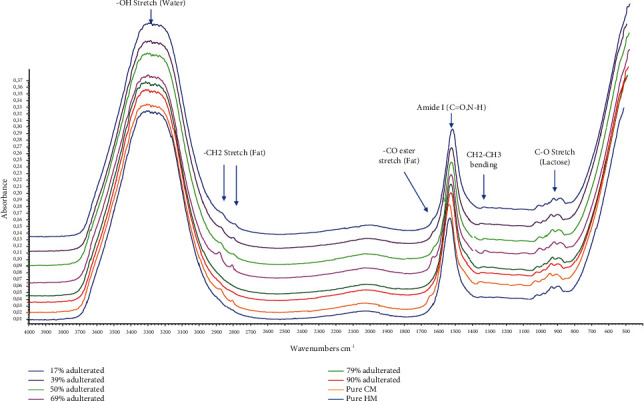
FTIR spectra of the binary mixture were selected from pure horse's milk (HM), cow's milk (CM), and adulterated milk (HM-CM) in the infrared region (4000-650 cm^−1^).

In this study, FTIR spectroscopy in the midinfrared region (4000-650 cm^−1^) combined with chemometrics of multivariate calibration and supervised pattern recognition of linear discriminant analysis (LDA) determined authentication analysis of HM from CM and GM. FTIR spectra are considered fingerprint tools for analytical purposes, including to assess milk authenticity, due to specific peaks and shoulders indicating functional groups presented in the valuated samples.  Figure 2 reveals FTIR spectra of milk, namely, horse milk (HM), cow milk (CM), goat milk (GM), and ternary mixture milk which had similar features. The identification of functional groups of these milk spectra is shown in Table 2. However, three spectra of HM, CM, and GM were distinguished from peak intensities as fingerprint property. These differences could be exploited as regions to optimize for chemometrics analysis. The peak at wavenumbers (1/*λ*) of 3320 cm^−1^ was due to -OH stretching vibration coming from water contents of milk in Figures 3 and 4. The wavenumbers of 1700-1500 cm^−1^ corresponded to amide groups (amide I and amide II) as specific in proteins and nucleic acids. Specifically, absorption peaks presented characterized the amide bands at 1635 cm^−1^ and 1455 cm^−1^ [20]. These peaks also were optimized during LDA and multivariate calibrations [21].

**Figure 5:**
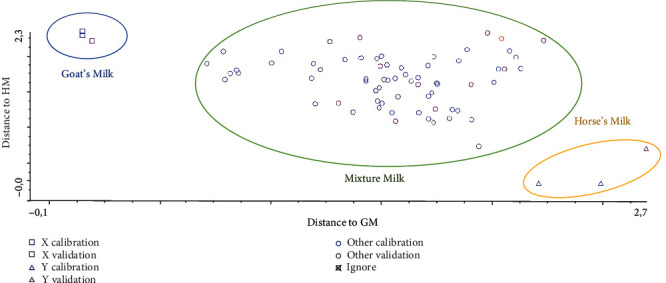
Cooman's plot for discrimination between horse's milk (*Δ*), goat's milk (□), and horse's milk adulterated with goat's milk (o).

**Figure 6:**
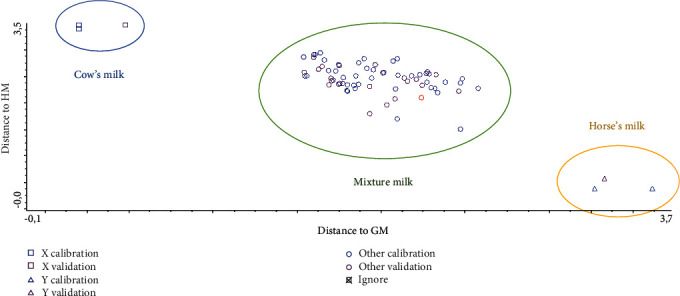
Cooman's plot for discrimination between horse's milk (*Δ*), cow's milk (□), and horse's milk adulterated with cow's milk (o).

Linear discriminant analysis (LDA) is one of the supervised pattern recognition techniques, which is commonly used for the discrimination of two or more objects (samples). In this study, LDA is applied to predict the class membership of unknown samples (HM and HM adulterated with CM and GM) based on the measurements of FTIR spectra at certain wavenumber regions as variables [22]. The absorbance values at certain finger regions of 1500-1000 cm^−1^ were used as variables and then converted to Mahalanobis distance for grouping HM and HM adulterated with CM to form Cooman's plot. From Figures 5 and 6, it is clear that both groups are separated clear with no classification objects observed. This indicated that LDA was successful for the discrimination of authentic HM from CM (A) and GM (B) as milk adulterants. Misclassification may occur because of the close similarities in chemical composition among groups or the inappropriate selection of wavenumbers [23].

**Figure 7:**
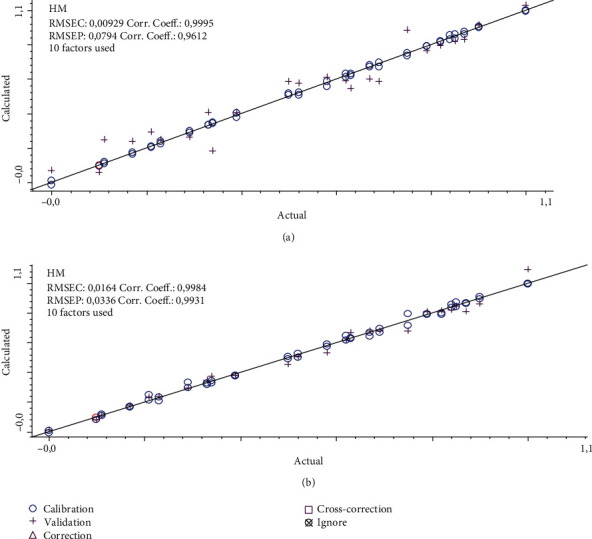
The correlation between the actual value of goat milk (a) and cow milk (b) with FTIR predicted values facilitated by partial least square calibrations.

The quantification of milk adulterants was facilitated with the use of multivariate calibrations of partial least square regression (PLSR) and principal component regression (PCR). FTIR spectra were subjected for spectral preprocessing, namely, Savitzy-Golay derivatization (1^st^ derivative and 2^nd^ derivative). Normal and derivative FTIR spectra at certain wavenumber regions combined with multivariate calibrations were compared to get the best model for the prediction of CM as an adulterant. Derivatization of FTIR spectra could improve the resolution of adjacent peaks which may affect the better performance of calibration modeling; however, the higher order of spectra derivative could decrease the model sensitivity [24]. The statistical parameters used as criteria were the coefficient of determination (*R*^2^) between actual values and FTIR predicted values for accuracy evaluation, as well as root mean square error of calibration (RMSEC) and root mean square error of prediction (RMSEP) for evaluation of precision (Tables 3 and 4). The selection of FTIR spectral condition was based on its capability to provide high *R*^2^ and low values of RMSEC and RMSEP [25]. Based on the optimization, PLSR using 2^nd^ derivative spectra at the combined wavenumber region of 3200-2800 and 1300-1000 cm^−1^ provided the best model for the relationship between actual values of goat milk (GM) and FTIR predicted values than PCR. At this condition, *R*^2^ values for calibration and validation models obtained were 0.9995 and 0.9612 with RMSEC and RMSEP values of 0.0093 and 0.0794, respectively. This result indicated that the combination of FTIR spectra and PLSR could be an effective method for the prediction of GM as an adulterant in HM with accurate and precise results as indicated by high *R*^2^ values and low RMSEC and RMSEP values.  Figure 7(a) reveals the relationship between actual values of GM and FTIR predicted values using the optimum condition.

PLSR using normal FTIR spectra at combined wavenumber region of 3800-3000 and 1500-1000 offered *R*^2^ for a relationship between actual values of CM and FTIR predicted values of 0.9984 for calibration and 0.9931 for validation models with low errors of RMSEC of 0.0164 and RMSEP of 0.0336 during authentication of HM from CM. These results suggested that FTIR spectroscopy in combination with LDA and PLSR is an effective means for authentication of HM from GM and CM. The close relationship between actual values of CM (*x*-axis) and FTIR predicted values (*y*-axis) existed (Figure 7(b)) meaning that the PLSR method is adequate to detect and predict the level of GM and CM in HM samples. From the residual analysis, it is clearly obtained that errors occurring during PLSR modeling of GM and CM as adulterants were negligible because no systematic errors were observed [26]. The residual analysis also demonstrated that there is no outlier data observed in the model.

Infrared spectroscopy predicted the fatty acid profile along with other milk components according to the vibration of functional groups [21]. Discriminating technique coupled with spectroscopy method could monitor for preselection of milk suppliers from adulteration. Moreover, FTIR spectra using the optimized condition could also provide accurate and precise results for the prediction of GM and CM as adulterants in HM. From the above results, it suggested that FTIR spectroscopy combined with chemometrics could be used as a fast analytical technique for authentication of horse milk from its adulterants (GM and CM). It offers simplicity in sample preparation because it can be used for direct authentication of fresh milk samples and green analytical technique due to less solvent requirement and provides high reproducibility. Additionally, this method is also less expensive than the gas chromatography (GC) technique as the common method for milk authentication through fatty acid analysis. Despite its advantages, this method also has drawbacks such as the developed model is only suitable for the same type of samples. It means that for different samples with different matrices, a new calibration model is required to be developed. In any case, the FA profiling using GC-FID is more validated than FTIR spectra to investigate different geographic origins [27]. According to the advantages and disadvantages of the developed method, this method could be used as a promising method for milk authentication because the advantages outweigh the disadvantages.

## 4. Conclusion

FTIR spectra in combination with chemometrics of linear discriminant analysis (LDA) were successfully applied for the classification between authentic horse milk (HM) and HM adulterated (GM and CM) without any misclassification observed. In addition, PLSR could provide the quantitative analysis of adulterants (GM and CM) reliably. The developed method is a fast and green analytical technique because it avoids the use of chemicals and solvents.

## Figures and Tables

**Table 1:** The binary mixture of samples containing horse's milk and goat's milk/cow's milk.

Sample	Horse's milk	Goat's milk/cow's milk
1	10	90
2	17	83
3	63	37
4	33	67
5	62	38
6	82	18
7	50	50
8	39	61
9	87	13
10	79	21
11	21	79
12	58	42
13	69	31
14	23	77
15	85	15
16	52	48
17	11	89
18	67	33
19	75	25
20	84	16
21	90	10
22	29	71
23	34	66
24	100	0
25	0	100

**Table 2:** IR absorption of common bands detected in milk.

Region	Wavenumber	Functional group
Water	3320 cm^−1^	-OH stretching vibrations
Fatty acid	2922 cm^−1^	-CH_2_ asymmetric stretching
	2852 cm^−1^	-CH_2_ symmetric stretching
	1742 cm^−1^	C=O from an ester of triacylglycerol
	1462 cm^−1^	CH_2_-CH_3_ bending vibrations
Protein	1635 cm^−1^	C=O stretching vibrations N-H bending vibrations
Lactose	1077 cm^−1^	C-O stretching vibrations

**Table 3:** The optimization wavenumber region of multivariate calibration for authentication of horse's milk in binary mixture with goat's milk.

Wavenumber (cm^−1^)	Multivariate calibration	Spectra	Calibration		Prediction	
			RMSEC	*R* ^2^	RMSEP	*R* ^2^
1500-900	PLS	Normal	0.0252	0.9961	0.0284	0.9954
		1^st^ derivative	0.0293	0.9948	0.0381	0.9922
		2^nd^ derivative	0.0252	0.9961	0.0632	0.9763
	PCR	Normal	0.0300	0.9945	0.0284	0.9954
		1^st^ derivative	0.0358	0.9922	0.0459	0.9884
		2^nd^ derivative	0.0620	0.9763	0.0797	0.9618
1800-1000	PLS	Normal	0.0979	0.9399	0.1120	0.9251
		1^st^ derivative	0.0094	0.9995	0.0383	0.9930
		2^nd^ derivative	0.0087	0.9995	0.0739	0.9672
	PCR	Normal	0.0323	0.9937	0.0355	0.9941
		1^st^ derivative	0.0399	0.9903	0.0602	0.9809
		2^nd^ derivative	0.0709	0.9690	0.0888	0.9559
3500-2800	PLS	Normal	0.0705	0.9693	0.0796	0.9666
		1^st^ derivative	0.1040	0.9325	0.1420	0.8778
		2^nd^ derivative	0.1600	0.8296	0.2380	0.5642
	PCR	Normal	0.1100	0.9237	0.1070	0.9386
		1^st^ derivative	0.1930	0.7393	0.2140	0.6713
		2^nd^ derivative	0.2370	0.5607	0.2820	0.2386
3000-2800	PLS	Normal	0.0602	0.9777	0.0894	0.9527
		1^st^ derivative	0.0580	0.9793	0.0883	0.9529
		2^nd^ derivative	0.1130	0.9191	0.1440	0.8659
	PCR	Normal	0.0735	0.9666	0.0889	0.9525
		1^st^ derivative	0.0993	0.9381	0.1270	0.8987
		2^nd^ derivative	0.2100	0.6808	0.2340	0.5775
3200-2800 and 1300-1000	PLS	Normal	0.0474	0.9863	0.0500	0.9859
		1^st^ derivative	0.0248	0.9962	0.0417	0.9903
		**2** ^**nd**^ **derivative** ^∗^	**0.0093**	**0.9995**	**0.0794**	**0.9612**
	PCR	Normal	0.0592	0.9784	0.0643	0.9775
		1^st^ derivative	0.0471	0.9864	0.0554	0.9829
		2^nd^ derivative	0.1470	0.8596	0.1380	0.8971

^∗^The selection condition was assigned with bold.

**Table 4:** The optimization wavenumber region of multivariate calibration for authentication of horse's milk (HM) from cow's milk (CM).

Wavenumber (cm^−1^)	Multivariate calibration	Spectra	Calibration		Prediction	
			RMSEC	*R* ^2^	RMSEP	*R* ^2^
1500-900	PLS	Normal	0.0345	0.9928	0.0315	0.9943
		1^st^ derivative	0.0476	0.9861	0.0500	0.9856
		2^nd^ derivative	0.0177	0.9981	0.0838	0.9571
	PCR	Normal	0.0415	0.9895	0.0349	0.9928
		1^st^ derivative	0.0543	0.9819	0.0522	0.9851
		2^nd^ derivative	09.1180	0.9121	0.1280	0.9021
3800-3000 and 1500-1000	PLS	**Normal** ^∗^	**0.0164**	**0.9984**	**0.0336**	**0.9931**
		1^st^ derivative	0.0079	0.9996	0.0743	0.9733
		2^nd^ derivative	0.2760	0.2697	0.2960	0.0097
	PCR	Normal	0.0658	0.9733	0.0614	0.9776
		1^st^ derivative	0.0921	0.9471	0.1020	0.9587
		2^nd^ derivative	0.2470	0.5108	0.2670	0.3692
3800-3000 and 2000-700	PLS	Normal	0.0447	0.9878	0.0466	0.9867
		1^st^ derivative	0.0474	0.9863	0.0639	0.9793
		2^nd^ derivative	0.0436	0.9884	0.1180	0.9264
	PCR	Normal	0.0618	0.9765	0.0583	0.9793
		1^st^ derivative	0.0805	0.9597	0.0759	0.9723
		2^nd^ derivative	0.1710	0.8026	0.1960	0.7894
3800-3000 and 2100-700	PLS	Normal	0.0442	0.9881	0.0455	0.9874
		1^st^ derivative	0.0466	0.9867	0.0659	0.9779
		2^nd^ derivative	0.0428	0.9888	0.1210	0.9231
	PCR	Normal	0.0633	0.9753	0.0598	0.9785
		1^st^ derivative	0.0816	0.9587	0.0773	0.9720
		2^nd^ derivative	0.1740	0.7936	0.2010	0.7689
3400-3000 and 1500-700	PLS	Normal	0.0652	0.9739	0.0592	0.9787
		1^st^ derivative	0.0299	0.9948	0.0550	0.9867
		2^nd^ derivative	0.0329	0.9934	0.1630	0.8353
	PCR	Normal	0.0678	0.9717	0.0621	0.9765
		1^st^ derivative	0.0599	0.9779	0.0753	0.9725
		2^nd^ derivative	0.2530	0.4696	0.2720	0.3229
3600-3000 and 2000-900	PLS	Normal	0.0347	0.9926	0.0421	0.9894
		1^st^ derivative	0.0079	0.9996	0.0570	0.9834
		2^nd^ derivative	0.0430	0.9887	0.1010	0.9451
	PCR	Normal	0.0558	0.9809	0.0541	0.9824
		1st derivative	0.0765	0.9638	0.0729	0.9728
		2^nd^ derivative	0.1220	0.9044	0.1530	0.8852

^∗^The selection condition was assigned with bold.

## Data Availability

All data used to support the findings of this study are included within the article.

## References

[1] Davoodi S. H., Shahbazi R., Esmaeili S. (2016). Health-related aspects of milk proteins. *Iranian Journal of Pharmaceutical Research*.

[2] Ballard O., Morrow A. L. (2013). Application of the SMART2 model to a forested catchment in Finland: comparison to the human milk composition: nutrients and bioactive factors. *Pediatric Clinics of North America*.

[3] Sharifi Rad J., Hoseini Alfatemi M., Sharifi Rad M. (2013). Horse milk: the composition, equine milk proteins, milk allergy and homology between mammal species with horse. *British Biomedical Bulletin*.

[4] Wu R., Chen J., Zhang L., Wang X., Yang Y., Ren X. (2021). LC/MS-based metabolomics to evaluate the milk composition of human, horse, goat, and cow from China. *European Food Research and Technology*.

[5] Esteki M., Shahsavari Z., Simal-Gandara J. (2020). Gas Chromatographic Fingerprinting Coupled to Chemometrics for Food Authentication. *Food Reviews International*.

[6] Abbas O., Zadravec M., Baeten V. (2018). Analytical methods used for the authentication of food of animal origin. *Food Chemistry*.

[7] Windarsih A., Rohman A., Riyanto S. (2021). The combination of vibrational spectroscopy and chemometrics for analysis of milk products adulteration. *International Journal of Food Science*.

[8] Hao X., Fu L., Shao L. (2022). Quantification of major milk proteins using ultra-performance liquid chromatography tandem triple quadrupole mass spectrometry and its application in milk authenticity analysis. *Food Control*.

[9] Xia Y., Yu J., Miao W., Shuang Q. (2020). A UPLC-Q-TOF-MS-based metabolomics approach for the evaluation of fermented mare's milk to koumiss. *Food Chemistry*.

[10] Nardiello D., Natale A., Palermo C., Quinto M., Centonze D. (2018). Milk authenticity by ion-trap proteomics following multi-enzyme digestion. *Food Chemistry*.

[11] Nardiello D., Natale A., Palermo C., Quinto M., Centonze D. (2017). Combined use of peptide ion and normalized delta scores to evaluate milk authenticity by ion-trap based proteomics coupled with error tolerant searching. *Talanta*.

[12] Eisenstecken D., Stanstrup J., Robatscher P., Huck C. W., Oberhuber M. (2021). Fatty acid profiling of bovine milk and cheese from six European areas by GC-FID and GC-MS. *International Journal of Dairy Technology*.

[13] Farah J. S., Cavalcanti R. N., Guimarães J. T. (2021). Differential scanning calorimetry coupled with machine learning technique: an effective approach to determine the milk authenticity. *Food Control*.

[14] Zain S. M., Behkami S., Bakirdere S., Koki I. B. (2016). Milk authentication and discrimination via metal content clustering - a case of comparing milk from Malaysia and selected countries of the world. *Food Control*.

[15] da Paixao Teixeira J. L., dos Santos Carames E. T., Baptista D. P., Gigante M. L., Pallone J. A. L. (2020). Vibrational spectroscopy and chemometrics tools for authenticity and improvement the safety control in goat milk. *Food Control*.

[16] Lucio-Gutiérrez J. R., Coello J., Maspoch S. (2011). Application of near infrared spectral fingerprinting and pattern recognition techniques for fast identification of Eleutherococcus senticosus. *Food Research International*.

[17] Liu N., Parra H. A., Pustjens A., Hettinga K., Mongondry P., van Ruth S. M. (2018). Evaluation of portable near-infrared spectroscopy for organic milk authentication. *Talanta*.

[18] Du L., Lu W., Gao B., Wang J., Yu L. L. (2019). Authenticating raw from reconstituted milk using Fourier transform infrared spectroscopy and chemometrics. *Journal of Food Quality*.

[19] Nicolaou N., Xu Y., Goodacre R. (2010). Fourier transform infrared spectroscopy and multivariate analysis for the detection and quantification of different milk species. *Journal of Dairy Science*.

[20] Rohman A., Windarsih A., Lukitaningsih E., Rafi M., Betania K., Fadzillah N. A. (2020). The use of FTIR and Raman spectroscopy in combination with chemometrics for analysis of biomolecules in biomedical fluids: a review. *Biomedical Spectroscopy and Imaging*.

[21] Balan B., Dhaulaniya A. S., Jamwal R. (2020). Application of attenuated total reflectance-fourier transform infrared (ATR- FTIR) spectroscopy coupled with chemometrics for detection and quantification of formalin in cow milk. *Vibrational Spectroscopy*.

[22] Messai H., Farman M., Sarraj-Laabidi A., Hammami-Semmar A., Semmar N. (2016). Chemometrics methods for specificity, authenticity and traceability analysis of olive oils: principles, classifications, and applications. *Food*.

[23] Rohman A., Che Man Y. B. (2009). Monitoring of virgin coconut oil (VCO) adulteration with palm oil using Fourier transform infrared spectroscopy. *Journal of Food Lipids*.

[24] Fadzlillah N. A., Che Man Y. B., Rohman A. (2014). FTIR spectroscopy combined with chemometric for analysis of sesame oil adulterated with corn oil. *International Journal of Food Properties*.

[25] Rohman A., Man Y. B. C., Ali M. D. E. (2019). The authentication of virgin coconut oil from grape seed oil and soybean oil using FTIR spectroscopy and chemometrics. *International Journal of Applied Pharmaceutics*.

[26] Jamwal R., Amit, Kumari S. (2020). Attenuated total reflectance-Fourier transform infrared (ATR-FTIR) spectroscopy coupled with chemometrics for rapid detection of argemone oil adulteration in mustard oil. *Lebensmittel-Wissenschaft und Technologie*.

[27] Bergamaschi M., Cipolat-Gotet C., Cecchinato A., Schiavon S., Bittante G. (2020). Chemometric authentication of farming systems of origin of food (milk and ripened cheese) using infrared spectra, fatty acid profiles, flavor fingerprints, and sensory descriptions. *Food Chemistry*.

